# Preliminary clinical study of personalized neoantigen vaccine therapy for microsatellite stability (MSS)-advanced colorectal cancer

**DOI:** 10.1007/s00262-023-03386-7

**Published:** 2023-02-16

**Authors:** Yao-Jun Yu, Na Shan, Li-Yi Li, Yue-Sheng Zhu, Li-Miao Lin, Chen-Chen Mao, Ting-Ting Hu, Xiang-Yang Xue, Xiao-Ping Su, Xian Shen, Zhen-Zhai Cai

**Affiliations:** 1 Department of Gastrointestinal Surgery, The Second Affiliated Hospital of Wenzhou Medical University, Wenzhou, 325000 People’s Republic of China; 2 Department of Gastroenterology, Shaoxing People’s Hospital, Shaoxing, 312000 People’s Republic of China; 3 Department of Gastroenterology, The Second Affiliated Hospital of Wenzhou Medical University, Wenzhou, 325000 People’s Republic of China; 4 Department of Gastroenterology, The First Affiliated Hospital of Wenzhou Medical University, Wenzhou, 325000 People’s Republic of China; 5 School of Basic Medicine, Wenzhou Medical University, Wenzhou, 325000 People’s Republic of China; 6 Department of Gastrointestinal Surgery, The First Affiliated Hospital of Wenzhou Medical University, Wenzhou, 325000 People’s Republic of China; 7 Department of General Surgery, The Second Affiliated Hospital & Yuying Children’s Hospital of Wenzhou Medical University, Wenzhou, Zhejiang Province People’s Republic of China

**Keywords:** Colorectal cancer, Immunotherapy, MSS, Neoantigen, Personalized vaccine

## Abstract

Immunotherapy based on immune checkpoint inhibitors (ICIs) has provided revolutionary results in treating various cancers. However, its efficacy in colorectal cancer (CRC), especially in microsatellite stability-CRC, is limited. This study aimed to observe the efficacy of personalized neoantigen vaccine in treating MSS–CRC patients with recurrence or metastasis after surgery and chemotherapy. Candidate neoantigens were analyzed from whole-exome and RNA sequencing of tumor tissues. The safety and immune response were assessed through adverse events and ELISpot. The clinical response was evaluated by progression-free survival (PFS), imaging examination, clinical tumor marker detection, circulating tumor DNA (ctDNA) sequencing. Changes in health-related quality of life were measured by the FACT-C scale. A total of six MSS–CRC patients with recurrence or metastasis after surgery and chemotherapy were administered with personalized neoantigen vaccines. Neoantigen-specific immune response was observed in 66.67% of the vaccinated patients. Four patients remained progression-free up to the completion of clinical trial. They also had a significantly longer progression-free survival time than the other two patients without neoantigen-specific immune response (19 vs. 11 months). Changes in health-related quality of life improved for almost all patients after the vaccine treatment. Our results shown that personalized neoantigen vaccine therapy is likely to be a safe, feasible and effective strategy for MSS–CRC patients with postoperative recurrence or metastasis.

**Supplementary Information:**

The online version contains supplementary material available at 10.1007/s00262-023-03386-7.

## Introduction

Colorectal cancer (CRC) is one of the most common malignant tumors in the world, accounting for approximately 1.2 million diagnoses and over 600,000 direct or indirect deaths each year [1]. The prognosis of advanced CRC is poor, and the current regimens mainly consist of surgical resection, chemoradiotherapy, and targeted therapy [2].The majority of the patients with metastatic colorectal cancer (mCRC) lose the opportunity of resection surgery at the initial diagnosis, while several problems have been encountered in the selection of the treatment population and the course of disease in chemoradiotherapy and targeted therapy [3, 4].There is no doubt that immunotherapy is now a standard treatment along with surgery, chemotherapy, radiotherapy and targeting therapy; and the field of cancer immunotherapy is continuing to develop [5]. In 2017, immune checkpoint inhibitors (ICIs) were approved for clinical use in patients with microsatellite instability-high (MSI-H) CRC. However, only 5% of advanced CRC patients with MSI-H can benefit from ICIs therapy. Most advanced CRC patients with microsatellite stability (MSS) fail to benefit from this treatment [6–8]. Therefore, there is an urgent need to develop a novel immunotherapy strategy for patients with MSS-CRC and to facilitate personalized treatment of MSS–CRC.

Tumor vaccines play an important role in amplifying the anti-tumor immune response in cancer patients. Neoantigens are tumor-specific antigens that arise due to somatic mutations in the tumor genome. Neoantigens possess significant immunogenic potential as they are not expressed in normal tissues. Because of their strong affinity for major histocompatibility complex (MHC), neoantigen-based therapy can be recognized by the host's immune system as non-self, which would prevent them from inducing central and peripheral immune tolerance mechanisms [9, 10]. Personalized vaccines have achieved therapeutic effects in a variety of tumors. In July 2017, two clinical studies demonstrated the successful treatment of advanced melanoma based on personalized neoantigens [11, 12]. Maria et al. identified neoantigen-reactive tumor-infiltrating lymphocytes (TILs) in patients with common gastrointestinal tumors; they also identified the immunogenicity of gene products encoded by somatic nonsynonymous mutations based on in vitro T-cell recognition assays [13]. Eric Tran et al. identified a polyclonal CD8 + T-cell response against mutant KRAS G12D in tumor-infiltrating lymphocytes obtained from a patient with metastatic colorectal cancer [14].These findings demonstrate that neoantigen vaccines may hold great potential for the treatment of a variety of tumors, including colorectal cancer. Our previous study confirmed the effectiveness of the neoantigen vaccine in a mouse CRC model [15].However, the role of neoantigens in MSS–CRC patients remains unclear.

In this study, we conducted a clinical research of neoantigen vaccines for MSS–CRC patients with recurrence or metastasis after surgery and chemotherapy and explored the identification and effective screening of neoantigens. Our results preliminarily demonstrate the feasibility, safety and immunological activity of the neoantigen vaccine, which is expected to bring a major breakthrough in treating patients with MSS–CRC, prolonging the progression-free survival time, and improving the treatment and quality of life of these patients.

## Patients and methods

### Study oversight

This study is a single-arm, single-center clinical study conducted at the Second Affiliated Hospital of Wenzhou Medical University in China and was registered at the Chinese Clinical Trial Registry (http://www.chictr.org.cn/; ChiCTR1900022372; The registration date was April 8, 2019). The corresponding design, protocol, and modification of this study, involving human samples, were approved by the Medical Ethics Committee of the Second Affiliated Hospital of Wenzhou Medical University (MEC numbers: LCKY2018-67). The protocols were strictly performed according to the Helsinki Declaration, and the study adhered to the privacy rights of humans. All enrolled patients signed a written informed consent form. The clinical trial was conducted following the agreement and its revised scheme, and all authors can view the experimental data and ensure the accuracy and integrity of the data and analysis.

### Patients

The patients were included based on the following inclusion criteria: (1) diagnosed with CRC, with TNM stages of IIIb–IV; (2) 18 to 75 years old male or female, with at least one radiographically measurable lesion; (3) patients had tumor recurrence or metastasis after surgery and chemotherapy, and their life expectancy should be at least 3 months; and (4) serum bilirubin not higher than 1.5 times the upper limit of normal (ULN), ALT or AST not higher than 2.5 times ULN, and creatinine clearance higher than 50 mL/min. For chemotherapy and targeted drug therapy, patients were required to have a washout period of at least 2 weeks.

Patients were excluded from this study based on the following criteria: (1) patients with HIV infection, HCV infection, serious coronary artery disease, or other diseases deemed unsuitable for inclusion in this study by the researchers; (2) patients with a history of bone marrow or organ transplantation; (3) patients with coagulation dysfunction; (4) drug abuse, alcohol abuse, clinical or psychological or social factors affecting informed consent or study implementation; (5) patients with secondary brain metastasis; and (6) any uncertain factors affecting the safety or compliance of patients.

### Neoantigen identification

#### Sample collection and DNA/RNA preparation

All tumor tissues were obtained from paraffin sections retained after surgery (patients 1, 2, 3, 4, 5, 6, 7, 8, 9, 10, and 11). Blood samples were collected and mixed with an EDTA-based anticoagulant. The extraction of DNA and RNA was performed from the paraffin sections of the postoperative tumor tissues with the All Prep DNA/RNA Mini Kit (Qiagen, Valencia, CA, USA/Illumina, San Diego, CA, USA); DNA from EDTA-anti coagulated peripheral blood samples was extracted with the QIAamp DNA Blood Mini Kit (Qiagen).

#### Whole-exome DNA and RNA sequencing

DNA sequencing was performed by generating two groups of double-ended sequencing results, corresponding to the collected tumor and blood cells (normal samples), in FASTQ format in Illumina HiSeq X10 platform (paired-end run, 150 bp). Fastp (Fastp v0.16.0) was used to control the quality of the original sequencing data and filter the low-quality data. Burrows-Wheeler Aligner (BWA-MEM, v.0.7.17-r1188) was used to compare the filtered clean reads with GRCh38/hg38 human reference genome to generate the sequence alignment map (SAM) file. Two groups of BAM files were obtained by utilizing SAMtools (v1.7–2) to convert SAM files into binary alignment map (BAM) files and establish indexes. The Genome Analysis Toolkit (GATK, v.4.1.3.0) was utilized for the removal of the variations from the DNA sequencing results.

For RNA sequencing, terminal sequencing results, corresponding to the collected tumor tissues, in FASTQ format were generated in Illumina HiSeq X10 platform (paired-end run, 150 bp). Fastp (Fastp v0.16.0) was utilized to control the quality of RNA sequencing data and filter low-quality data. The STAR software (2.6.0a) was utilized to compare the filtered clean reads with GRCh38/hg38 human reference genome to generate a SAM file. Based on SAMtools (V1.7), the SAM file was converted into a BAM file, and indexes were established. The Genome Analysis Toolkit (GATK, v.4.1.3.0) was utilized for detecting the variations in the RNA sequencing results.

#### Human leukocyte antigen (HLA) typing

Based on the DNA sequencing data, patients’ HLA class I genotypes (HLA-A, HLA-B, and HLA-C) were assessed using the OptiType software. Based on the RNA clean reads, HLA class II genes (DQA1, DQB1, DRB1, DRA, DPA1, and DPB1) encoding MHC class II receptors were analyzed based on Seq2HLA (V 2.3).

#### Neoantigen filtering

Integration analysis of DNA mutations and HLA was conducted based on MuPeXI (version 1.2.0) [16], NetMHCpan 4.0, and the variant effect predictor (VEP) (version ensembl-vep-release-92) databases [17]. The HLA-binding affinity scores (IC50) were obtained for all 8–11-mer variants for MHC class I molecules and 12–15-mer variants for MHC class II molecules. Expression level of mutant genes was determined from RNA-seq data in transcripts per million. Each peptide was given a priority score on the basis of HLA-binding affinity, expression level, similarity to self-peptides, and mutant allele frequency. Peptides with a priority score larger than 0 were selected as neoantigen candidates. Neoantigen candidates with high priority score will be finally selected to synthetic vaccine.

### MSI testing

MSI sensor scans a given reference genome to find the locations of homologous polymers and microsatellites. In this study, homopolymers of at least 5-bp length and microsatellites of maximum repeat unit length of five were recorded from the reference genome, and the location and flanking sequence of each site were saved in a site file for subsequent analysis. A standard* χ*^2^ test was performed at each site having at least 20 reads in both the tumor and normal samples to assess the goodness-of-fit between their respective *k*-mer distributions (Sokal and Rohlf, 2012). For each sample, the total number of sites with sufficient data (at least 20 cross reads both in normal and tumor) and the number of somatic sites were noted; the percentage of somatic sites gave the score of MSI.

### Personalized neoantigen long‑peptide vaccine synthesis and vaccination

The personalized neoantigen peptides were synthesized by the standard solid-phase synthetic peptide chemistry on CS 536 peptide synthesizer. The polypeptide is 27 amino acids long, with the mutation site as the center, and 13 amino acids are added before and after the polypeptide [18, 19]. The purification and purity analysis of the polypeptide were performed by reversed phase-high performance liquid chromatography (RP-HPLC) (> 98% purity, endotoxin concentration was less than 0.01 EU/g and trifluoroacetic acid (TFA) residue was less than 1%).The quality inspection of the vaccine was carried out through the detection of bacterial endotoxin, fungal D-glucan, bacterial and fungal smear, and acute toxicity test in mice. Then, the neoantigen vaccine pools were mixed with 0.5 mg poly I:C (polyinosinic:polycytidylic acid) (Guangdong South China Pharmaceutical Co., Ltd.) and subcutaneously injected into both axilla and groin. Patients received the neoantigen vaccine on days 1, 4, 8, 15, and 22 (priming phase) and at weeks 12 and 20 (boosting phase).

### Safety assay and changes in health-related quality of life

Safety was assessed by an evaluation of the clinical adverse events, which were graded according to the Common Terminology Criteria for Adverse Events (CTCAE) (version 4.0) published by the National Cancer Institute (NCI). In addition, the changes in blood routine, urine routine, liver and kidney functions, and electrolyte and coagulation functions before and after vaccination were also employed as an objective for safety evaluation.

Changes in health-related quality of life were measured by the Functional Assessment of Cancer Therapy-Colorectal cancer (FACT-C) scale (version 4.0) which has been well-tested in cancer survivors and found to be reliable, valid, and responsive [20].

### Clinical tumor response monitoring

To complete clinical tumor marker detection, sufficient blood samples were collected at multiple time points before and after neoantigen vaccine therapy. Patients were evaluated for measurable tumor metastases with an enhanced CT scan every 2 months.

### Interferon-gamma (IFN-γ) enzyme-linked immunospot (ELISpot) assay

IFN-γ ELISPOT kit (Shenzhen Dakwei Biotechnology Co., Ltd, Anshan, China) was used to perform the ELISpot assay for detection of IFN-γ in neoantigen-specific T cells. Peripheral blood mononuclear cells (PBMC) separation was performed with 10 mL of peripheral blood, while dendritic cells (DC) and T cells were separated by a 6-well plate culture. Thereafter, DC and T cells were mixed in a ratio of 10:1 and aliquoted into 96-well plates for co-stimulation culture; 200 µL Rosewell Park Memorial Institute medium (RPMI 1640) containing 100 U/mL IL-2, 10% fetal bovine serum, and 4 µg polypeptide was added to each well. The co-stimulated cells were then transferred to the Elispot plate. While 1/1000 CD3 and phosphate-buffered saline (PBS) solution were applied to the positive control and negative control wells, 4 µg polypeptide was added to the polypeptide well. The cells were grown in a CO_2_ incubator at 37 °C for 24 h. The cells were then rinsed, and 100 µL of fresh medium containing 1 µg/mL 7-B6-1-biotin was added to each well before incubating for 2 h at room temperature. Cells in each well were rinsed five times with PBS buffer before being incubated with 100 µL of 3, 3’,5,5’-tetramethylbenzidine (TMB) color-developing solution. Once spots in the well were visible, the color-developing reaction was stopped by rinsing with water.

### Detection of the neoantigen mutation in cfDNA

Cell-free DNA (cfDNA) was extracted from serial plasma samples of the enrolled patients using QIAamp Circulating Nucleic Acid Kit (Qiagen). Detection of neoantigen mutation in cfDNA was performed as in the previous study with some changes [21]. We designed one reverse primer with variations in its 3′ nucleotide and one forward primer (Supplementary Table 1) for each neoantigen mutation. Polymerase chain reaction (PCR) amplifications were performed using the LC480 SYBR Green mix (Roche Diagnostics, Meylan, France). The cycling conditions were as follows: denaturation for 10 min at 95 °C; amplification for 45 cycles, with denaturation for 10 s at 95 °C, annealing for 15 s at 65 °C, and extension for 20 s at 72 °C.

### Statistical analysis

Data from all enrolled patients received at least 5 planned prime phase vaccinations were included in the safety and clinical outcome evaluation. Descriptive statistics were used to determine safety of neoantigen vaccine and changes in health-related quality of life. Bar chart of immune response and ctDNA dynamic curves were obtained using GraphPad Prism 8.

## Results

### Study design and patient characteristics

**Fig. 1:**
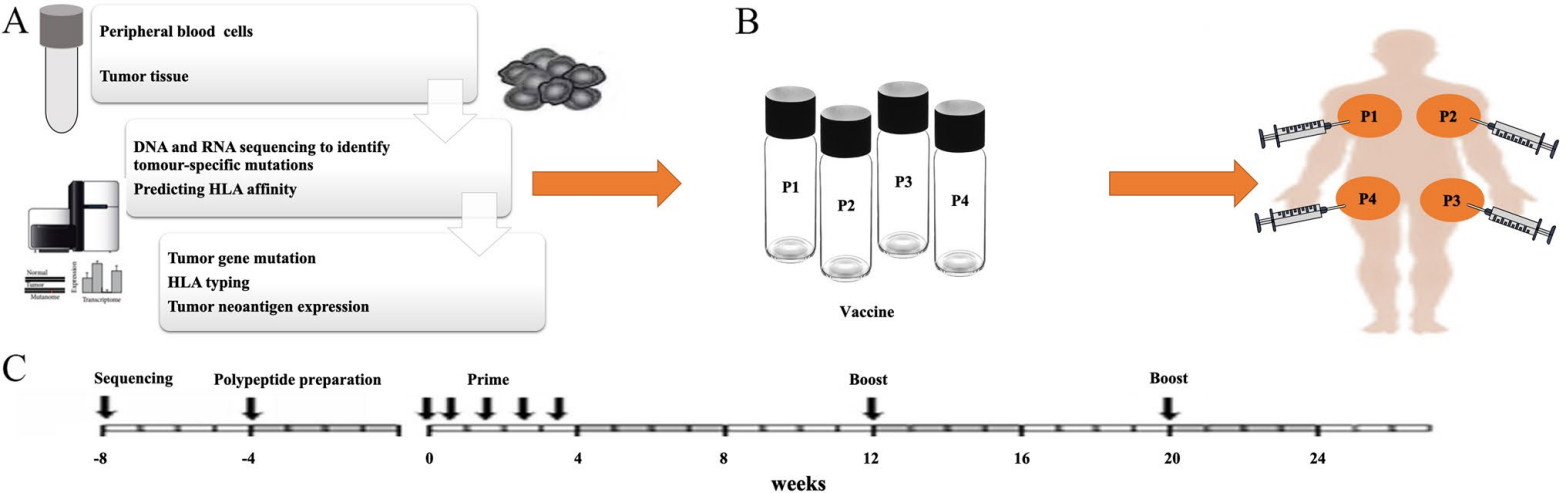
Vaccine preparation and injection schedule. **a** Tumor neoantigen analysis. The flow chart illustrates the whole process of tumor neoantigen analysis. **b** Personalized vaccination. Personalized peptide vaccines and polyinosinic-polycytidylic acid injection were co-injected subcutaneously. Each injection was subcutaneously administered in the bilateral axilla and bilateral groin. **c** Vaccine administration. The patients received the neoantigen peptide vaccine at days 1, 4, 8, 15, and 22 (the initial phase), and weeks 12 and 20 (the enhancement phase), respectively. The black arrows indicate the time points at which the injection was scheduled

This single-arm and single-center clinical trial evaluated the safety and feasibility of personalized neoantigen vaccines for the treatment of MSS–CRC patients with postoperative recurrence or metastasis. In this study, the safety and feasibility of personalized neoantigen vaccines were the primary endpoints, and the corresponding immune response and progression-free survival (PFS) were the secondary endpoints. This study analyzed the personalized neoantigens based on the whole-exome sequencing and transcriptome sequencing data of tumor tissue sections and peripheral blood samples retained after the operation (Fig. 1a). The neoantigen vaccine was mixed with poly I:C and subcutaneously injected into the patients' axilla and groin regions (Fig. 1b). According to the injection treatment plan, the priming-phase injections were administered on days 1, 4, 8, 15, and 22, whereas the boosting-phase injections were administered on weeks 12 and 20 (Fig. 1c).

After preliminary screening and clinician evaluation, 11 patients with postoperative recurrence or metastasis of CRC were initially included in the neoantigen screening. The postoperative tumor pathological tissues provided by four patients (patients W04, W05, W08, and W11) were screened out as they exhibited serious RNA degradation due to their long retention time, and the measured RNA data could not be used for the analysis of neoantigens. Also, patient W07 was screened out because the measured neoantigens were less consistent with the RNA transcription data. Finally, a total of 6 CRC patients with postoperative recurrence or metastasis were included to evaluate the safety and efficacy of neoantigen vaccine. After MSI detection, it was found that all 6 patients were MSS–CRC. The demographics and baseline clinical characteristics of the six patients are listed in Table 1.

**Table 1:** Baseline clinical characteristics of the patients

Patient ID	Age	Sex	Cancer diagnosis	Prior therapy	Tumor stage	Intervals between surgery and vaccination	Distant metastases	Tumor differentiation	MSI
W01	45	M	Rectal	Surgery, L-OHP, 5-FU, CAPE, CPT-11, BEV	T4N2bM1c	9 months	Peritoneal metastasis	Low-medium differentiation	MSS
W02	58	M	Colon	Surgery, L-OHP, CAPE	T2N2aM1a	28 months	Liver metastasis	High-medium differentiation	MSS
W03	37	M	Colon	Surgery, MWA, BAI, BAE	T2N2aM1b	117 months	Liver and lung metastasis	Medium differentiation	MSS
W06	45	F	Colon	Surgery, MWA, CPT-11, 5-FU, BEV, Fruquintinib	T3N1bM1a	23 months	Liver metastasis	Medium differentiation	MSS
W09	43	F	Sigmoid colon	L-OHP, CAPE, Surgery, MWA, CPT-11, 5-FU, BEV	T3N1aM1a	19 months	Liver and lung metastasis	Medium differentiation	MSS
W10	72	M	Rectal	Surgery, L-OHP, CAPE, CPT-11, 5-FU, C225	T4N2aM0	20 months	Pelvic lymph nodes metastases	Medium differentiation	MSS

*M* male; *F* female; *L-OHP* oxaliplatin; *CAPE* capecitabine; *5-FU* 5-Fluorouracil; *CPT-11* Irinotecan; *BEV* bevacizumab; *C225* cetuximab; *BAI, BAE* bronchial artery infusion and embolization; *MWA* microwave ablation, *MSI* microsatellite instability; *MSS* microsatellite stability

**Fig. 2:**
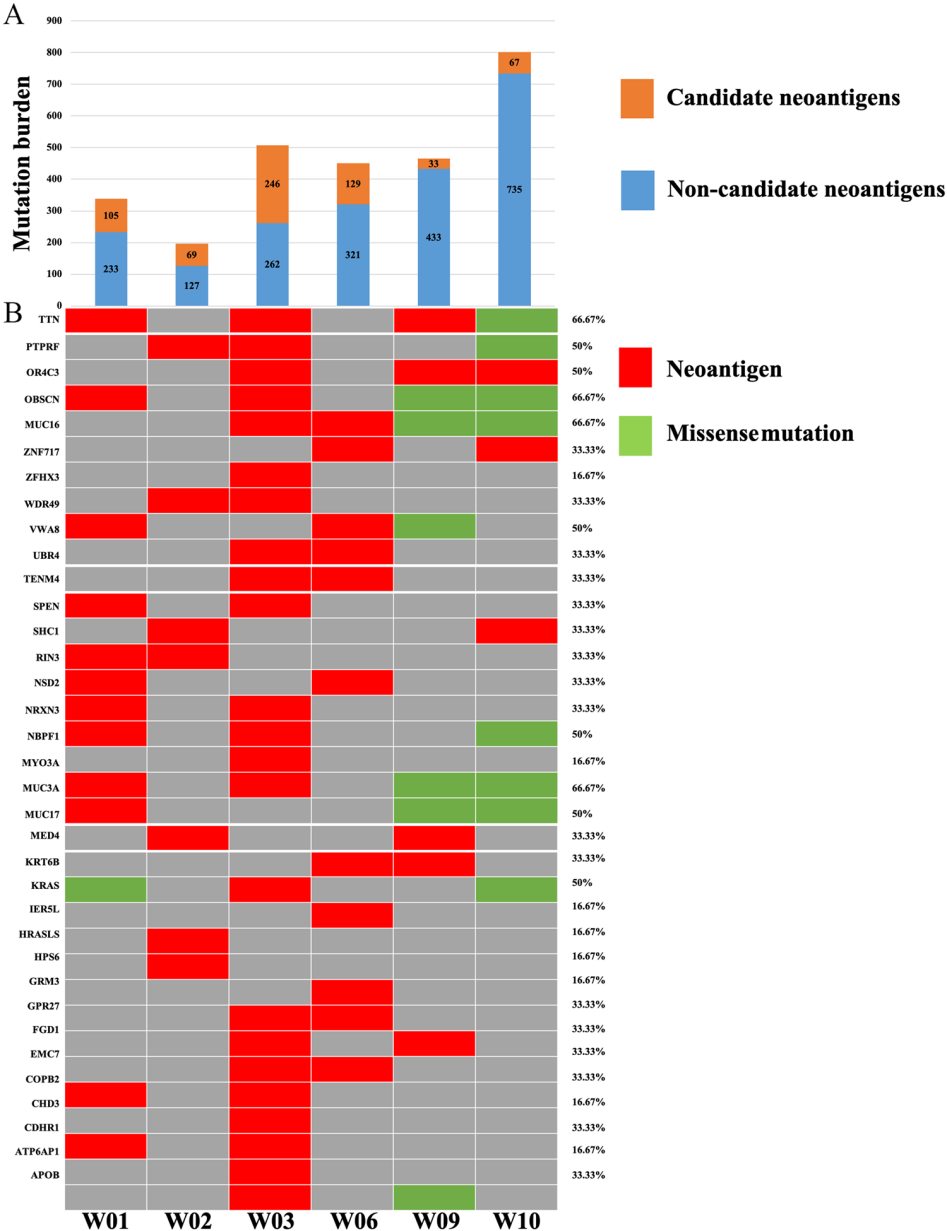
The overview of neoantigen profiles. **a** Number of candidate neoantigens (priority score > 0) and non-candidate neoantigens (priority score < 0) detected in each of the six CRC patient's tissue samples. **b** Heatmap summarizing the somatic/neoantigen hotspot gene profile in the enrolled CRC patients

A total of 3192 nonsynonymous somatic single-nucleotide variants (SNVs) were identified based on whole-exome sequencing of the tumor and matched peritumor tissues, with an average of 456 mutations (range 196–802) in each patient. Only 29.2% (range 7.08–48.43%) of these somatic mutations were confirmed for expression by transcriptome sequencing analysis and identified as neoantigens by epitope prediction (Fig. 2a). Of all the candidate neoantigens, only the neoantigen with high priority score can be finally selected to synthetic vaccine. For each patient, 7–19 personalized neoantigen long-peptides derived from somatic point mutations were synthesized for vaccine manufacture. The neoantigens finally selected to synthetic vaccine for 6 patients are detailed in Supplementary Table 2. It was observed that 66.67% (4/6) of patients had neoantigens derived from TTN, OBSCN, MUC16, and MUC3A mutations. But even if different patients have neoantigens derived from the same mutations, their specific mutation sites are different. We also performed statistical analysis for mutations with a frequency greater than 33.33% (2/6) (Fig. 2b). The results showed that these neoantigens emerged with high frequency often had low priority score, and only a few were selected for vaccine preparation.

### The safety of neoantigen vaccine and changes in health-related quality of life

Since there was a drug washout period of at least 2 weeks between vaccine treatment and conventional treatment, we could distinguish the clinical adverse reactions after vaccine treatment from those of conventional treatment. All six enrolled patients received five planned priming phase vaccinations. As for booster vaccinations, patients W01, W02, W03, and W10 received four, seven, three, and two booster vaccinations, respectively. No obvious treatment-related adverse events were observed, and routine blood/biochemical tests did not indicate any obvious abnormalities during vaccinations. After vaccination, all patients had mild injection-site reaction (Grade 1). Patient W02 experienced minor itching on his back following the third vaccination treatment, and patient W09 experienced minor headache following the first vaccination (Table 2). These symptoms went away quickly without treatment and did not reoccur during the follow-up injection therapy.

**Table 2:** Treatment-related adverse events in the patients

	All treated patients (*n* = 6)	
	Grade 1–2	Grade 3–4
*Constitutional*		
Injection-site reaction	6 (100%)	0
Flu-like symptoms	0	0
Fever	0	0
Fatigue	0	0
Chills	0	0
Dizziness	0	0
*Gastrointestinal*		
Nausea	0	0
Constipation	0	0
Vomiting	0	0
Diarrhea	0	0
Dry mouth	0	0
*Respiratory*		
Cough	0	0
Dyspnea	0	0
*Laboratory*		
Anemia	0	0
Neutropenia	0	0
*Other*		
Rash	1(16.7%)	0
Headache	1(16.7%)	0

*Including all patients who received at least one dose of trial treatment

Changes in health-related quality of life were measured on the FACT-C as listed in Table 3. Results indicated that all patients had varying degrees of improvement in their quality of life.

**Table 3:** Quality of life at baseline and at 20 weeks (*n* = 6)

		All treated patients (*n* = 6)											
		Baseline						20 weeks					
		W01	W02	W03	W06	W09	W10	W01	W02	W03	W06	W09	W10
FACT-C (0–132)		94	92	93	96	110	102	94	97	92	101	109	101
Physical well-being (0–28)		25	25	23	19	28	26	22	23	23	19	27	25
Social/family well-being (0–24)		14	15	16	19	17	15	15	19	16	21	17	17
Emotional well-being (0–24)		20	16	16	22	23	21	17	16	16	19	21	20
Functional well-being (0–28)		18	16	17	18	23	21	20	16	18	19	24	21
CRC subscale (0–28)		17	20	21	18	19	19	20	23	19	23	20	18

### Clinical and immune response monitoring during vaccination and follow-up

**Fig. 3:**
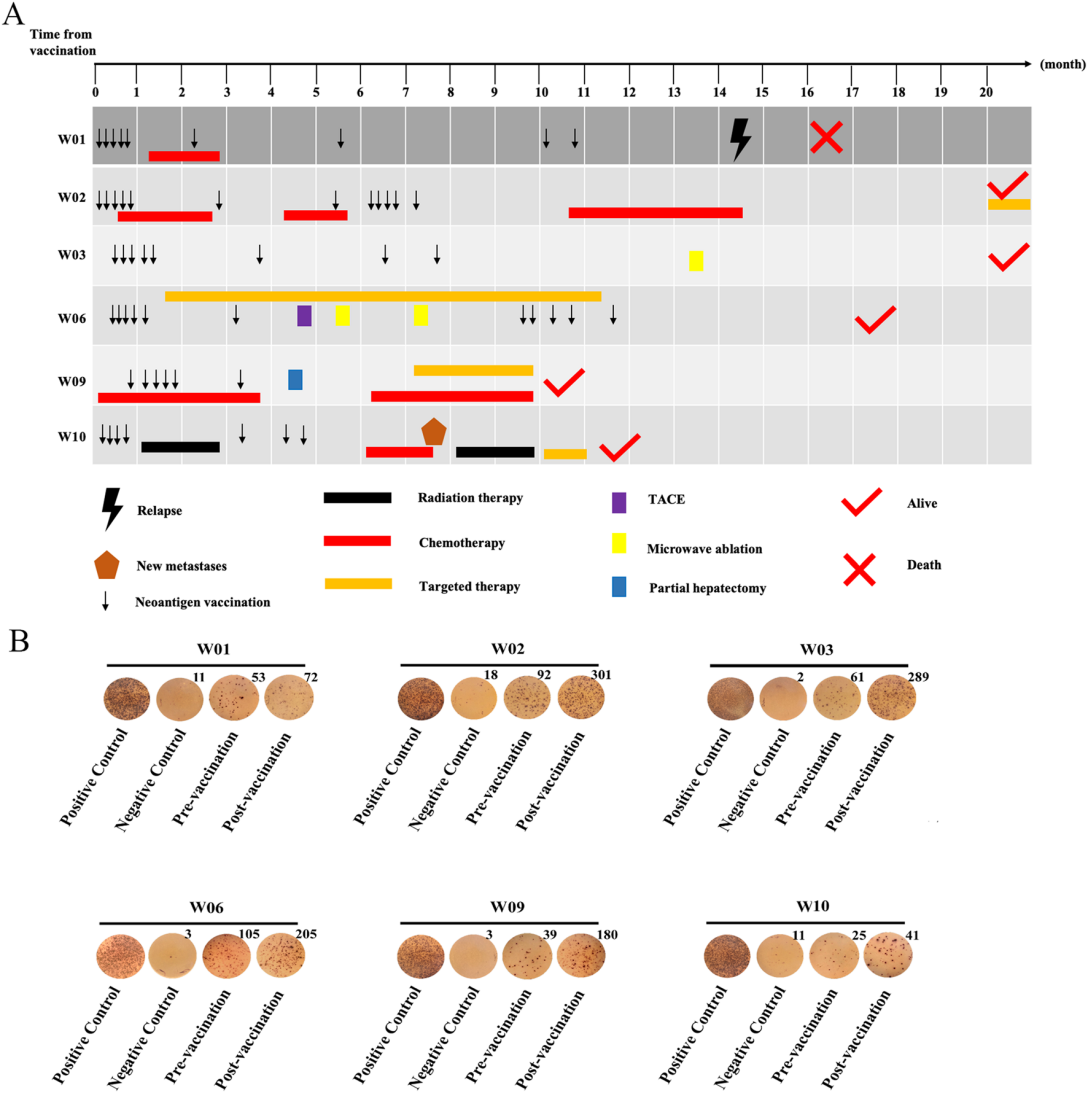
Clinical outcome and immune response monitoring in the enrolled CRC patients during vaccination and follow-up. **a** The detailed timeline presentation of clinical treatments, vaccinations, and clinical outcomes for six enrolled CRC patients from the beginning of vaccine treatment until the deadline of the clinical trial. **B** The ex vivo IFN-γ enzyme-linked immunoblot (ELISPOT) responses for PBMCs stimulated by personalized neoantigen pools before and after the neoantigen vaccination

The detailed timeline presentation of clinical treatments, vaccinations, and clinical outcomes is shown in Fig. 3a. The median follow-up time of six patients was 17 months (range 11–24 months). During the clinical trial, two of the six patients were confirmed with clinical relapse by pathological biopsy or MRI/CT scan, with a median PFS of 11 months; the other four patients remained relapse-free. (Mean follow-up time was 19 months.) After 14 months of follow-up, patient W01 developed a new tumor in the intestinal wall, which was pathologically confirmed as colonic adenocarcinoma. After 8 months of follow-up, the patient W10's spinal metastasis was confirmed by MRI/CT scan. Patient W03 is resting at home and being closely monitored among the four patients who have not progressed in their disease; patients W02, W06 and W09 are currently undergoing chemotherapy combined with targeted therapy.

During vaccination and follow-up, in vitro IFN-γ ELISpot assay was performed to monitor immune response by using autologous PBMCs stimulated by neoantigen pools or each neoantigen individually. IFN-γ ELISpot assay indicated that our standard vaccination protocol is efficient in the induction of antigen-specific immune response in CRC patients. As shown in Fig. 3b, the neoantigen-specific activation of PBMCs induced by neoantigen vaccines was observed in four out of six patients after vaccination. The ELISpot results showed that the neoantigen-reactive T cells (NRTs) in patients W02, W03, W06 and W09 are significantly more than that in patients W01 and W10 (Fig. 3b). Importantly, the PFS of these patients (Patients W02, W03, W06 and W09) was significantly longer than that of the other two (Patients W01 and W10) (19 vs 11 months).

## Discussion

**Fig. 4:**
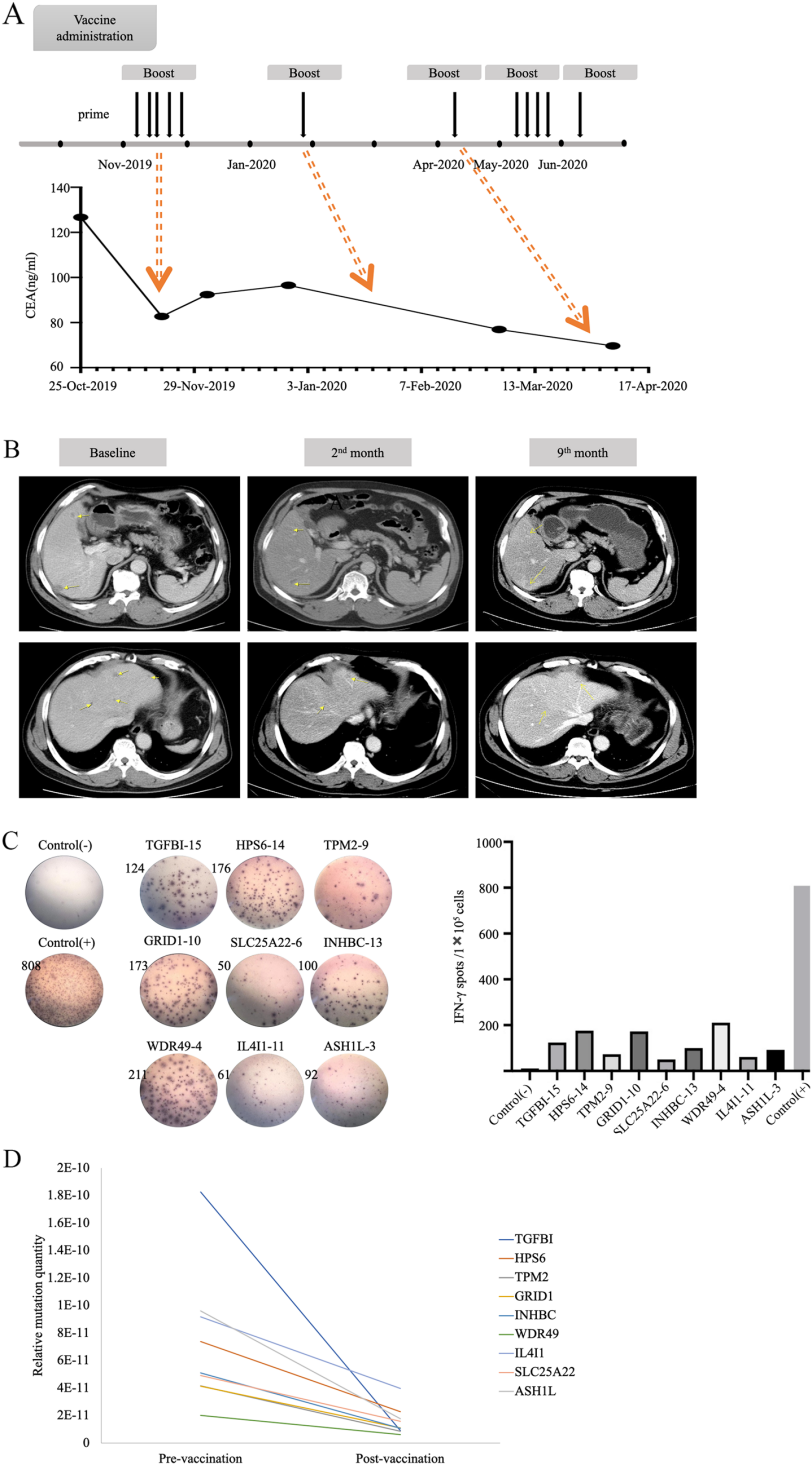
Case report of advanced colorectal cancer patient W02. **a** Clinical vaccine treatment timeline and corresponding tumor marker of patient W02. **b** Enhanced CT findings of liver metastases during the neoantigen vaccination and follow-up. **c** Monitoring of ex vivo IFN-γ ELISPOT response for PBMCs stimulated by personalized neoantigen in patient W02. **d** The performances of ctDNA for CRC monitoring in patient W02

This clinical trial was a single-arm, single-center study, which included six MSS–CRC patients with postoperative recurrence or metastasis. After the personalized neoantigen vaccination, four of six patients exhibited a positive immune response to the neoantigen vaccine and had significantly longer PFS time than the other two patients with negative neoantigen response (19 vs 11 months). No significant adverse events were observed in the six enrolled patients during the neoantigen vaccine administration. All patients reported that the quality of life had improved during the vaccine treatment. According to the analysis of the above results, even MSS–CRC patients with postoperative recurrence or metastasis are expected to benefit from this immunotherapy. The most surprising thing was the performance of patient W02. The patient received chemotherapy again when liver metastasis occurred after colorectal cancer surgery during which time there was no significant decrease in carcinoembryonic antigen (CEA). The patient started the single-drug treatment of vaccine after stopping the chemotherapy treatment. After four injections, the patient's CEA dropped from an initial 126.7 to 82.73 ng/mL (Fig. 4a). Abdominal-enhanced CT images at three different time points before and after treatment demonstrated that multiple space-occupying liver lesions decreased or even disappeared in varying degrees during the treatment (Fig. 4b). The IFN-γ spots per 10^5^ PBMCs of the peptide or peptide pool with the best response are shown in Fig. 4c. As shown in Fig. 4d, all monitored personalized nonsynonymous somatic mutations decreased to varying degrees after receiving the vaccine treatment.

In China, the incidence rate of CRC is fifth among all malignancies [22]; about 20% of CRC is first diagnosed at stage IV, and its 5-year survival rate is only about 13% [23–25]. At present, immunotherapy based on ICIs has been proven to be successful in treating lung cancer, metastatic melanoma, esophageal cancer, and other tumors [26–28]. Keynote-177 randomized phase III clinical trial demonstrated that PFS of patients with MSI-H metastatic CRC using pembrolizumab was better than chemotherapy. The imaging-based evaluation indicated that the total and complete remission rates of the pembrolizumab group were higher [29], thereby reinforcing the clinical value of immunization therapy based on ICIs in treating MSI-H metastatic CRC. In 2015 and 2016, the American Society of Clinical Oncology (ASCO) published clinical trials of programmed cell death protein 1 (PD-1)/programmed death-ligand 1 (PD-L1) inhibitors, pembrolizumab, and nivolumab in the treatment of advanced CRC. It was observed that the objective remission rate of MSI-H CRC patients was excellent; the remission time was long, and the adverse drug reactions were controllable. However, the vast majority of advanced patients with MSS do not benefit from ICIs-based immunotherapy [30]. Therefore, breaking the immune resistance of patients with MSS–CRC or proposing new immunotherapy methods has become a hot spot in the treatment of CRC. Our research included six MSS–CRC patients with postoperative recurrence or metastasis for the clinical trial of individualized tumor vaccine treatment. The preliminary results demonstrated that the personalized neoantigen vaccine is a safe, feasible, and effective strategy for MSS–CRC treatment and provided additional data on individualized medicine for MSS–CRC. Also, it can improve the quality of life of patients with MSS–CRC to a certain extent.

Previous studies had proved the safety and effectiveness of personalized tumor vaccines based on neoantigens in the treatment of melanoma and advanced lung cancer [18, 31]. Also, the literature indicated that vaccines based on individual mutations and predicted neo-epitopes could induce T-cell infiltration [32], which also proved the possibility of killing tumor cells by utilizing the autoimmune system. The existence of tumor-reactive CD8 (+) cells in gastrointestinal tumors had been confirmed at the molecular level, which also provided a basis for the development of immunotherapy for tumor patients [33]. Eric Tran et al. observed that TILs from nine out of ten patients with metastatic gastrointestinal cancers contained CD4( +) and/or CD8( +) T cells that recognized one to three neo-epitopes derived from somatic mutations expressed by the patient's own tumor [34]. Thus, a high frequency of patients with common gastrointestinal cancers harbor immunogenic mutations that can potentially be exploited for the development of highly personalized immunotherapies. Some clinical trials had proved the safety and effectiveness of vaccines based on tumor neoantigen [35]; however, there are few studies on tumor vaccine therapy in MSS–CRC. Importantly, our previous study found adoptive transfer of neoantigen-reactive T cells induced by vaccination with two mutant peptides could effectively inhibit tumor growth in tumor-bearing mouse models [15]. As tumors with a large number of mutations are linked to response to immunotherapy due to the larger number of potential neoantigens, we had a closer look at the number of neoantigens identified in six patients with MSS–CRC. Surprisingly, even patients with MSS–CRC have a considerable amount of neoantigens. Similar to the mutational rate, some studies had shown that the median number of neoantigens in CRC patients with MSI-H tumors was around 20 times higher than in MSS tumors [36].The amount of neoantigens may become a key factor in the benefit of tumor immunotherapy. However, the number of neoantigens selected for the personalized vaccine of patient W02 was the smallest among all patients, but the number of immune response cells in his IFN-γ ELISpot assay was the largest. We believe that the amount of neoantigens is not necessarily the only decisive factor to determine the benefit of immunotherapy. Screening neoantigens with strong immunogenicity for patients with MSS–CRC may greatly confer the greatest benefits in tumor immunotherapy. In our clinical trial, the four patients with great neoantigen-specific immune response had a significantly longer PFS than the other two patients without neoantigen-specific immune response (19 vs. 11 months). The strong neoantigen-specific immune response may be associated with longer PFS.

In summary, our results preliminarily prove that personalized neoantigen vaccine therapy is a safe, feasible and immunological effective strategy for MSS–CRC patients with postoperative recurrence or metastasis and may provide clinical benefit. Patients who exhibit stronger positive immune responses to the neoantigen vaccines tend to have better clinical outcomes. However, this study still has the following shortcomings: Firstly, we failed to detect the intensity of the immune response quantitatively and accurately determine the duration of the immune response; secondly, due to limited sample size enrolled in this study, the corresponding findings will still need to be validated in large-scale clinical trials.

## Supplementary Information

Below is the link to the electronic supplementary material.

Supplementary file1 (DOCX 16 KB)

Supplementary file2 (XLSX 11 KB)

## Data Availability

The data generated in this study are available within the article.
