# Supplementary material for: Preliminary clinical study of personalized neoantigen vaccine therapy for microsatellite stability (MSS)-advanced colorectal cancer
Source: Cancer Immunol Immunother. 2023 Feb 16;72(7):2045–56. doi: 10.1007/s00262-023-03386-7 (PMC10264512; doi:10.1007/s00262-023-03386-7)
Supplement: Supplementary file 1 — Supplementary file1 (DOCX 16 KB) [file 262_2023_3386_MOESM1_ESM.docx]

**Supplementary Table 1.** Primers for each neoantigen mutation.

| **Neoantigen** | **Forward primer** | **Reverse primer** |
| --- | --- | --- |
| TGFBI | TTTGACGTTACCAACTTCTCTTTTTCAGCC | AGTGCTTCAAGCTAATGCTTCATCCTCTCA |
| HPS6 | AGGTGAGGAGGGGACCAGGCCTGAGGCTCT | CTTGGAGCACAGCTTTAGGCCGCCCACTGT |
| TPM2 | AATCCTCAGCGATGTGCTTGGCCTCCTTCA | AAACCGGGCCATGAAGGATGAGGAGAAGAC |
| GRID1 | CTTCTGCCAGCGCAGCTCCGTCACCAGCCT | CCTACACACTGGCTTCGAGACCACCCGTCT |
| INHBC | GCATCTTGGACAAGCTGCACCTCACCCAGC | CAGTGCAGTCCTCAAAGCAGCTCTGGACAG |
| WDR49 | ACTTTTGGGGAAAGAACAAGCTATCCTCTG | TTTTCATCCAGGTTTTGAGACTCTGGGATG |
| IL4I1 | GGGCAGCGGCGGCGAGAAGGTGATGCGCTT | AGGTGCTGAAGGCCGACGTGGTGCTGCTGT |
| SLC25A22 | CACTCCCAGCCACACAGCCGGCCAGGAAGG | GAACCAGCTGGGCCGCCCGGCGTCCGAGGT |
| ASH1L | AGGATTACAGGCATGAGCGACCGTGCCCGG | ACTTAGAAATCTGTGGAAGGAATGGCACTG |

*The red mark in Reverse primer is the variant design.
